# Plasma multi-omics reveals pathogen-associated mechanisms and diagnostic signatures in *Escherichia coli* and *Klebsiella pneumoniae* bloodstream infections

**DOI:** 10.3389/fcimb.2026.1873717

**Published:** 2026-07-09

**Authors:** Yuxin Yang, Mei Yang, Yao Xian, Shunjie Yang, Jianpeng Su, Xue Chen, Xiaohong Feng, Fanglin Mao, Yan Kang, Qionglan Dong

**Affiliations:** 1 Department of Critical Care Medicine, West China Hospital, Sichuan University, Chengdu, China; 2 Department of Critical Care Medicine, The Third Hospital of Mianyang, Sichuan Mental Health Center, Mianyang, China

**Keywords:** bloodstream infection (BSI), *Escherichia coli*, *Klebsiella pneumoniae*, plasma multi-omics, sepsis

## Abstract

**Objective:**

*Escherichia coli* and *Klebsiella pneumoniae* are major Gram-negative pathogens causing bloodstream infection (BSI) and sepsis, but their plasma host-response patterns remain incompletely characterized. This study aimed to explore shared and pathogen-associated proteomic and metabolomic features in *E. coli* BSI and *K. pneumoniae* BSI.

**Methods:**

We performed plasma proteomic and metabolomic profiling in 100 participants, including patients with *E. coli* BSI (n = 26), *K. pneumoniae* BSI (n = 15), blood culture-negative sepsis (NegBC, n = 29), and healthy controls (HC, n = 30). Differential molecular analyses were performed with additional adjustment for baseline clinical covariates where applicable. Downstream GO, KEGG, PPI, and protein–metabolite network analyses were based on molecules that remained significant after covariate adjustment. A leakage-free internal validation framework was used to construct an exploratory plasma proteome-based model for distinguishing *E. coli* BSI from non-*E. coli* sepsis.

**Results:**

Both *E. coli* BSI and *K. pneumoniae* BSI showed prominent coagulation-immune alterations involving complement/coagulation-related pathways. *E. coli* BSI showed a more evident platelet-centered immunometabolic pattern, particularly relative to NegBC, whereas *K. pneumoniae* BSI showed coagulation-immune activation coupled with lipid and broader metabolic remodeling. In the direct comparison between *E. coli* BSI and *K. pneumoniae* BSI, no proteins remained significantly different after covariate adjustment, whereas metabolomic differences persisted, involving glycine, serine and threonine metabolism, terpenoid backbone biosynthesis, apoptosis, and folate biosynthesis. An exploratory *E. coli* BSI model based on FCGR3A, COPS3, NT5C3A, and ADO showed preliminary discrimination between *E. coli* BSI and non-*E. coli* sepsis in leakage-free internal validation (AUC = 0.806, 95% CI: 0.706–0.906).

**Conclusion:**

Plasma multi-omics suggested shared thromboinflammatory features and partially distinct platelet-associated and metabolic patterns in *E. coli* BSI and *K. pneumoniae* BSI. However, findings involving direct pathogen comparison and the *K. pneumoniae* BSI cohort should be interpreted cautiously because of baseline clinical heterogeneity and limited sample size. The candidate diagnostic model remains exploratory and requires external validation.

## 1 Introduction

Sepsis is a life-threatening organ dysfunction caused by a dysregulated host response to infection and remains a leading cause of mortality in intensive care units worldwide (Meyer and Prescott, 2024; La Via et al., 2025). Its high mortality is closely related to delayed diagnosis, delayed pathogen-directed treatment, and the complex interplay among immune, coagulation, endothelial, and metabolic disturbances that drive organ dysfunction [Girardis et al., 2024; Cao et al., 2023; Holmes et al., 2025]. Although advances in supportive care have improved management, the molecular basis of sepsis remains incompletely understood, particularly with respect to how different pathogens may shape distinct host response programs.

*Escherichia coli* and *Klebsiella pneumoniae* are among the most important Gram-negative pathogens causing BSI and subsequent sepsis [Pérez et al., 2024; Wong Fok Lung et al., 2022; Li et al., 2025]. Although both organisms belong to the Enterobacterales order and share several Gram-negative pathogen-associated molecular patterns, they differ substantially in virulence architecture and host-interaction strategies [Sora et al., 2021; Monteiro et al., 2024]. Extraintestinal pathogenic *E. coli* commonly expresses adhesins, siderophore systems, lipopolysaccharide, capsule-related structures, and toxins such as α-hemolysin, which may promote epithelial invasion, complement activation, platelet activation, and systemic inflammatory responses. In contrast, *K. pneumoniae* is characterized by prominent capsular polysaccharide-mediated immune evasion, hypermucoviscosity in hypervirulent strains, fimbrial adhesion, siderophore-mediated iron acquisition, and an increasing association with antimicrobial resistance. These pathogen-level differences may shape distinct host immune, coagulation, endothelial, and metabolic responses during BSI-associated sepsis.

In addition to their distinct virulence characteristics, these organisms may trigger different host responses and thereby imprint partially distinct proteomic and metabolic signatures on the septic state. While thromboinflammation and immunometabolic dysregulation are recognized hallmarks of sepsis [Nguyen et al.,2025; Palmowski et al.,2025], direct system-level comparisons between *E. coli*- and *K. pneumoniae*-associated BSI remain limited. Differentiating between *E. coli* and *K. pneumoniae* BSI is clinically relevant for several reasons. First, these pathogens may differ in dominant infection sources, virulence potential, antimicrobial-resistance patterns, and risks of invasive or complicated disease. Second, early pathogen-informed discrimination may help refine empirical antimicrobial strategies, risk stratification, and subsequent diagnostic prioritization before definitive microbiological results are available. Third, because sepsis has traditionally been studied as a pathogen-agnostic syndrome, direct comparison of *E. coli*- and *K. pneumoniae*-associated BSI may provide insight into whether common Gram-negative inflammatory responses are accompanied by pathogen-associated host-response signatures.

Blood culture remains the current diagnostic gold standard for BSI, but its clinical utility is limited by delayed turnaround time and reduced sensitivity after antimicrobial exposure [Dai et al., 2024]. This gap continues to drive empiric broad-spectrum treatment and highlights the need for pathogen-informative host-response biomarkers. In addition to healthy controls, blood culture-negative sepsis represents an important disease-control group because it captures the systemic inflammatory and metabolic background of clinically diagnosed sepsis without culture-confirmed bacteremia [Sigakis et al., 2019]. Including this group allows pathogen-associated molecular patterns in *E. coli* and *K. pneumoniae* BSI to be evaluated against both a non-septic baseline and a clinically relevant sepsis background, rather than only against healthy individuals.

To address these questions, we performed integrated plasma proteomic and metabolomic profiling in patients with *E. coli* BSI, *K. pneumoniae* BSI, blood culture-negative sepsis, and healthy controls. We aimed to identify shared and pathogen-associated host-response patterns, with particular attention to thromboinflammatory signaling and metabolic reprogramming. We also explored the feasibility of constructing an exploratory plasma proteome-based diagnostic model for *E. coli* BSI using an internal validation framework.

## 2 Materials and methods

### 2.1 Participant enrollment and grouping

This study was approved by the Ethics Committee of The Third Hospital of Mianyang (Approval No. 2024.13). Written informed consent was obtained from all participants or their legal representatives before enrollment.

From September 2024 to September 2025, patients meeting the Sepsis-3 criteria [Singer et al., 2016] were prospectively recruited at The Third Hospital of Mianyang. Sepsis was defined by suspected or confirmed infection together with an increase in Sequential Organ Failure Assessment (SOFA) score of at least 2 points. Participants were stratified into three sepsis groups according to blood culture results: the *E. coli* BSI group, the *K. pneumoniae* BSI group, and the blood culture-negative sepsis group (NegBC; no microbial growth after at least 5–7 days of standard incubation).

Exclusion criteria for patient groups were age <=18 years, pregnancy, >50% missing clinical data, advanced malignancy, severe immunodeficiency, or receipt of antibiotic or antiviral therapy within 1 month before admission or before blood sample collection. The healthy control (HC) group comprised age- and sex-matched adults (>18 years) without recent antibiotic or anti-infective drug exposure.

Collected clinical variables included demographic characteristics (age and sex), disease-severity indices (age-adjusted Charlson Comorbidity Index [aCCI], Acute Physiology and Chronic Health Evaluation II [APACHE II], and SOFA), laboratory parameters, septic shock, and major clinical outcomes.

Because this was an exploratory plasma multi-omics study aimed at identifying pathogen-associated host-response patterns, no formal *a priori* sample size calculation based on a prespecified effect size was performed. The final sample size was determined by the number of eligible participants enrolled during the study period, particularly under the strict requirement that blood samples had to be collected before antimicrobial administration. Therefore, analyses involving the K. pneumoniae BSI group were interpreted cautiously because of the limited sample size and were considered exploratory.

### 2.2 Plasma sample collection and processing

Peripheral venous blood samples were collected from patients with sepsis within 6 hours after diagnosis and before antimicrobial administration. Blood samples were collected into EDTA anticoagulant tubes and gently inverted to ensure adequate anticoagulation. To reduce cellular and platelet contamination, plasma was prepared using a two-step centrifugation protocol. Briefly, blood samples were first centrifuged at 600 g for 10 minutes at 4 °C to separate plasma from cellular components. The upper plasma fraction was then transferred to a new tube and centrifuged again at 2,500 g for 15 minutes at 4 °C to reduce residual platelet contamination. After the second centrifugation, the upper plasma fraction was carefully collected into sterile cryovials without disturbing the bottom pellet. Samples from healthy controls were collected and processed using the same protocol. Plasma aliquots were flash-frozen in liquid nitrogen and stored at -80 °C until analysis.

### 2.3 Plasma proteomic profiling

Plasma proteomic profiling was performed using LC-MS/MS in DIA mode. Briefly, plasma proteins were enriched with a commercial low-abundance protein enrichment kit and then digested with trypsin. For proteomic sample preparation, an equal volume of plasma (50 μL) was used for each sample. Protein quantification was not performed before enzymatic digestion. After digestion, peptides were quantified using NanoDrop-based peptide quantification, and equal peptide amounts were used for LC-MS/MS analysis. The resulting peptides were desalted using solid-phase extraction columns, separated on a C18 reversed-phase column, and analyzed on a timsTOF_HT mass spectrometer operating in DIA mode. Protein identification and quantification were performed with Spectronaut software (v15.7) against the human UniProt database. The false discovery rate (FDR) was controlled at <1% at both precursor and protein levels. Detailed chromatography and mass spectrometry parameters are provided in the **Supplementary Data**. During LC-MS/MS acquisition and initial proteomic data processing, operators were blinded to the clinical group allocation and clinical status of the participants. Sample grouping information was used only during downstream statistical analysis.

Raw protein intensity values were log2-transformed and median-normalized to reduce technical variation. Proteins with missing values in more than 50% of samples within any group were excluded. Remaining missing values were imputed using the k-nearest neighbors (KNN) algorithm. Differentially expressed proteins (DEPs) were defined using |log2(fold change)| >= 1 and adjusted *P* < 0.05 after Benjamini-Hochberg correction. For unadjusted two-group proteins comparisons, *P* values were calculated using a two-tailed Student’s t-test on log2-transformed normalized values. To reduce the influence of baseline clinical imbalance, covariate-adjusted analyses were further performed using linear models. For comparisons among sepsis groups, the models were adjusted for sex, APACHE II score, and lactate level. For comparisons involving healthy controls, only available demographic covariates, including sex, were adjusted because severity-related variables such as APACHE II score and lactate level were not applicable to healthy participants. DEPs that remained significant after covariate adjustment were used for downstream biological analyses. Gene Ontology (GO) annotation and Kyoto Encyclopedia of Genes and Genomes (KEGG) pathway enrichment analyses were performed using the clusterProfiler package in R. Protein-protein interaction (PPI) networks were constructed using the STRING database [Szklarczyk et al., 2021] and visualized in Cytoscape [Shannon et al., 2003].

### 2.4 Plasma metabolomic profiling

Untargeted metabolomic profiling was performed using LC-MS/MS [Cui et al., 2018]. Data were acquired in both positive (POS) and negative (NEG) ionization modes. All pre-analytical handling steps were conducted on dry ice. Metabolites were extracted using pre-cooled methanol:acetonitrile (3:1, v/v) containing 2,6-dichlorophenylalanine as an internal standard. Quality control (QC) samples were interspersed throughout the analytical sequence to monitor instrument stability. Detailed procedures for sample preparation and mass spectrometry are provided in the **Supplementary Data**.

Raw LC-MS/MS data were processed using Compound Discoverer 3.3 for peak detection, alignment, integration, and correction. Metabolite annotation was performed by matching acquired spectra against mzCloud, ChemSpider, KEGG, and HMDB, supplemented by a local mzVault spectral library. Orthogonal partial least squares-discriminant analysis (OPLS-DA) was used to visualize group separation, and model stability was assessed by 100 permutation tests. Differentially expressed metabolites (DEMs) were defined by a Variable Importance in Projection (VIP) score > 1.0, |log2(fold change)| > 0.585, and *P* < 0.05. For unadjusted two-group metabolites comparisons, *P* values were calculated using a two-tailed Student’s t-test on log2-transformed normalized values. Because the number of DEMs identified in the present dataset was limited, Benjamini-Hochberg adjusted *P* values were not used as the primary screening criterion for DEMs. Therefore, the possibility of false-positive findings cannot be excluded, and metabolomic findings were interpreted cautiously as exploratory and pathway-oriented results.

To reduce the influence of baseline clinical imbalance, covariate-adjusted analyses were further performed using linear models. For comparisons among sepsis groups, the models were adjusted for sex, APACHE II score, and lactate level. For comparisons involving HC group, only available demographic covariates, including sex, were adjusted. DEMs that remained significant after covariate adjustment were used for downstream biological analyses. Metabolic pathway enrichment analysis was conducted using KEGG, with *P* < 0.05 considered significant.

### 2.5 Model construction and internal validation

A leakage-free diagnostic modeling framework was used to explore plasma proteomic signatures for pathogen-associated BSI discrimination. The primary task was to distinguish *E. coli* BSI from non-*E. coli* sepsis, with *E. coli* BSI coded as the positive class and *K. pneumoniae* BSI plus blood culture-negative sepsis coded as the negative class. Healthy controls were excluded from diagnostic modeling to avoid capturing sepsis-versus-health differences. Because of the limited sample size, the *K. pneumoniae* BSI model was considered exploratory and reported only in the **Supplementary Material**.

Internal validation was performed using stratified five-fold cross-validation repeated 100 times. To prevent data leakage, candidate protein screening, Z-score standardization, LASSO feature selection, and logistic regression model fitting were performed entirely within each training fold. Candidate proteins were screened from all quantified proteins after adjustment for sex, APACHE II score, and lactate level, and the held-out test fold was used only to generate predicted probabilities. Feature stability was assessed by calculating the selection frequency of each protein across all cross-validation folds, and proteins with a selection frequency >= 0.50 were defined as relatively stable candidate biomarkers. Model discrimination was assessed using ROC curves and AUC. The optimal probability threshold was determined by maximizing the Youden index, and sensitivity, specificity, accuracy, positive predictive value, negative predictive value, and Youden index were calculated at this threshold.

### 2.6 Statistical analysis

**Figure 1:**
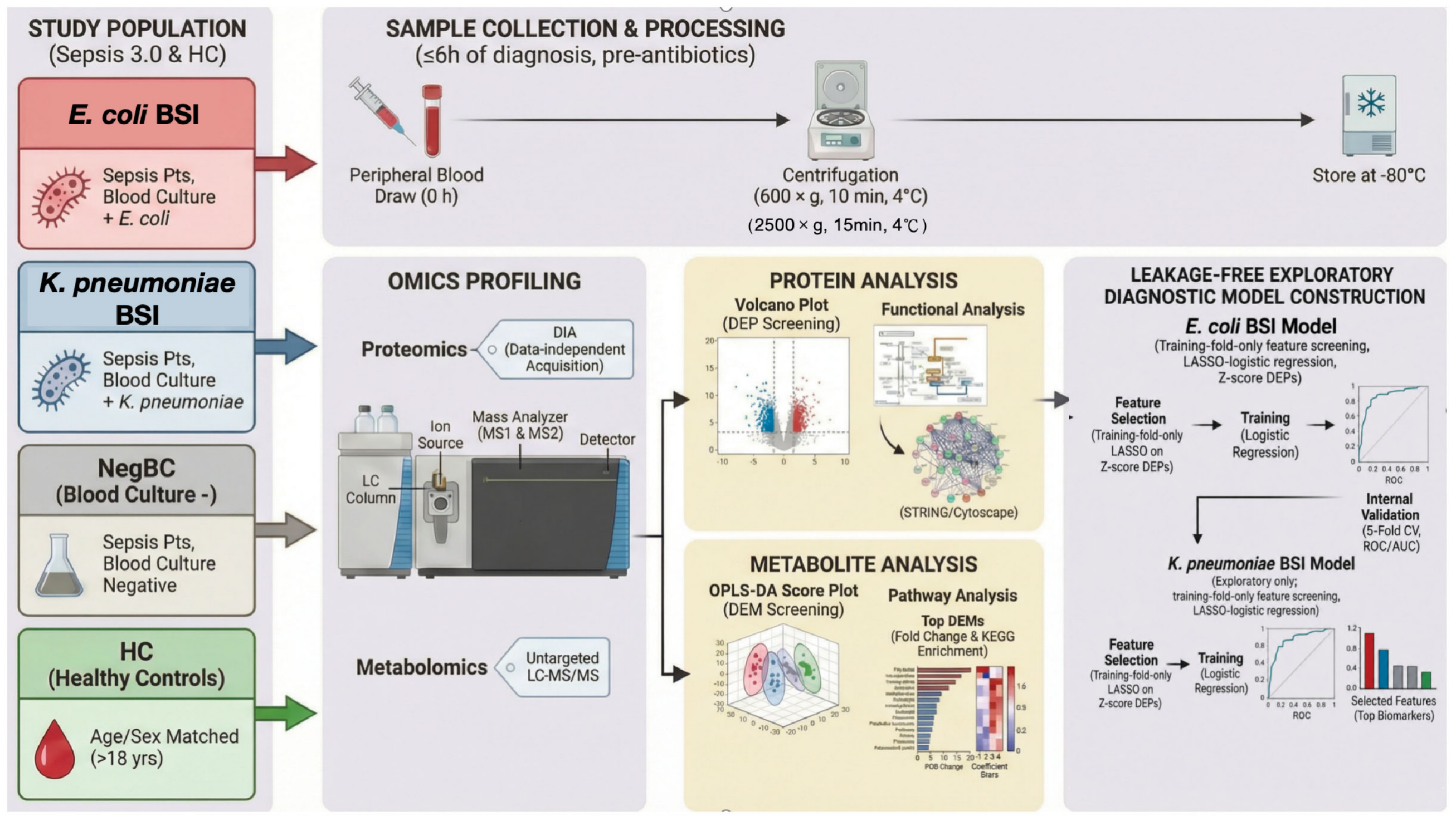
Overview of participant grouping, plasma sample collection, LC-MS/MS-based proteomic and metabolomic profiling, bioinformatics analysis, multi-omics integration, and diagnostic model construction.

Continuous variables are presented as median (interquartile range) and were compared among the three sepsis groups using the Kruskal-Wallis test. Categorical variables are expressed as number (percentage) and were compared using the chi-square test or Fisher’s exact test, as appropriate. All statistical analyses were performed using R software (version 4.3.2), and a two-sided *P* < 0.05 was considered statistically significant. *Post-hoc* pairwise comparisons, if necessary, were conducted using Dunn’s test with Bonferroni correction. The overall study workflow is summarized in **Figure 1**.

## 3 Results

### 3.1 Participant characteristics

A total of 100 participants were included, comprising 70 patients with sepsis (*E. coli* BSI, n = 26; *K. pneumoniae* BSI, n = 15; NegBC, n = 29) and 30 HC. Among the sepsis groups, age and aCCI did not differ significantly, whereas the *K. pneumoniae* BSI group showed a higher proportion of male patients and a higher APACHE II score. SOFA scores were not significantly different across groups (**Table 1**).

**Table 1:** Baseline characteristics of the patients.

Variables	*E. coli* BSI (n = 26)	*K. pneumoniae* BSI (n = 15)	NegBC (n = 29)	HC (n=30)	P
Age, years	69.00 (60.00,79.00)	70.00 (48.00,80.50)	67.00 (60.00,75.00)	68.00 (62.25,75.75)	0.849
Male, n (%)	9 (34.62)	13 (86.67)	16 (55.17)	18 (60.00)	0.013
aCCI	4.00 (3.00,5.75)	4.00 (3.00,6.00)	4.00 (2.00,5.00)	-	0.515
APACHE II Score	11.00 (8.00,17.75)	22.00 (15.50,25.50)	18.00 (15.00,23.00)	-	0.019
SOFA Score	6.50 (5.00,9.00)	8.00 (5.00,10.00)	9.00 (6.00,10.00)	-	0.427
Lac, mmol/L	4.20 (3.15,5.30)	5.60 (3.75,7.30)	2.70 (2.20,3.90)	-	0.001
P/F, mmHg	310.50 (248.50,370.50)	300.00 (198.50,353.00)	288.00 (202.00,341.00)	-	0.563
WBC, 10^9/L	11.86 (8.97,13.64)	9.12 (6.31,9.89)	11.84 (9.69,15.92)	-	0.029
Hct	33.00 (26.85,36.00)	30.20 (19.95,39.15)	32.10 (27.10,35.60)	-	0.724
Neutrophil, 10^9/L	10.77 (6.40,11.85)	6.16 (3.80,9.14)	11.00 (8.26,13.84)	-	0.045
Platelet, 10^9/L	126.00 (77.50,173.00)	111.00 (71.00,184.00)	120.00 (81.00,202.00)	-	0.960
ALT, U/L	23.00 (13.25,40.50)	52.00 (16.00,74.00)	28.00 (14.00,88.00)	-	0.480
AST, U/L	25.50 (19.00,50.75)	64.00 (29.00,108.50)	43.00 (20.00,139.00)	-	0.158
TBIL, μmol/L	17.55 (11.97,30.35)	15.40 (9.45,33.50)	13.80 (9.10,24.40)	-	0.595
DBIL, μmol/L	6.65 (4.88,15.10)	6.70 (3.95,23.55)	6.20 (3.70,14.40)	-	0.716
Cr, μmol/L	102.00 (64.50,176.75)	101.00 (71.00,142.00)	131.00 (90.00,241.00)	-	0.119
PT, sec	14.00 (12.70,15.75)	14.00 (12.05,18.70)	13.60 (11.90,15.90)	-	0.844
INR	1.19 (1.08,1.36)	1.32 (1.10,2.03)	1.22 (1.07,1.45)	-	0.619
D-D, μg/mL	2.93 (1.27,7.13)	4.63 (3.17,10.07)	5.32 (2.99,12.32)	-	0.033
FIB, g/L	4.88 (3.64,7.24)	2.93 (1.77,4.92)	4.74 (3.80,5.95)	-	0.050
PCT, ng/mL	33.93 (6.59,89.18)	17.27 (1.91,48.79)	19.29 (4.61,97.13)	-	0.560
IL-6, pg/mL	2769.53 (1431.00,3907.90)	1241.00 (452.10,3765.26)	2921.03 (560.79,4975.57)	-	0.267
Shock, n (%)	10 (38.46)	7 (46.67)	19 (65.52)	-	0.123

aCCI, age-adjusted Charlson Comorbidity Index; APACHE II, Acute Physiology and Chronic Health Evaluation II; SOFA, Sequential Organ Failure Assessment; Lac, lactate; P/F, arterial oxygen tension/inspired oxygen fraction; WBC, white blood cell count; Hct, hematocrit; ALT, alanine aminotransferase; AST, aspartate aminotransferase; TBIL, total bilirubin; DBIL, direct bilirubin; Cr, creatinine; PT, prothrombin time; INR, international normalized ratio; D-D, D-dimer; FIB, fibrinogen; PCT, procalcitonin; IL-6, interleukin-6.

### 3.2 Plasma multi-omics characteristics of *E. coli* BSI: emphasis on thromboinflammatory and platelet-centered immunometabolic networks

Quality control analyses supported the stability and reproducibility of the LC-MS/MS datasets. In the proteomic analysis, the HeLa external QC samples showed highly consistent pairwise correlations, supporting stable mass spectrometry performance during acquisition. The pooled plasma QC samples (QC_H) and commercial plasma standard samples (QC_S) also showed strong correlation consistency, indicating good reproducibility of the plasma proteomic workflow (**Supplementary Figures 1A–C**).

**Figure 2:**
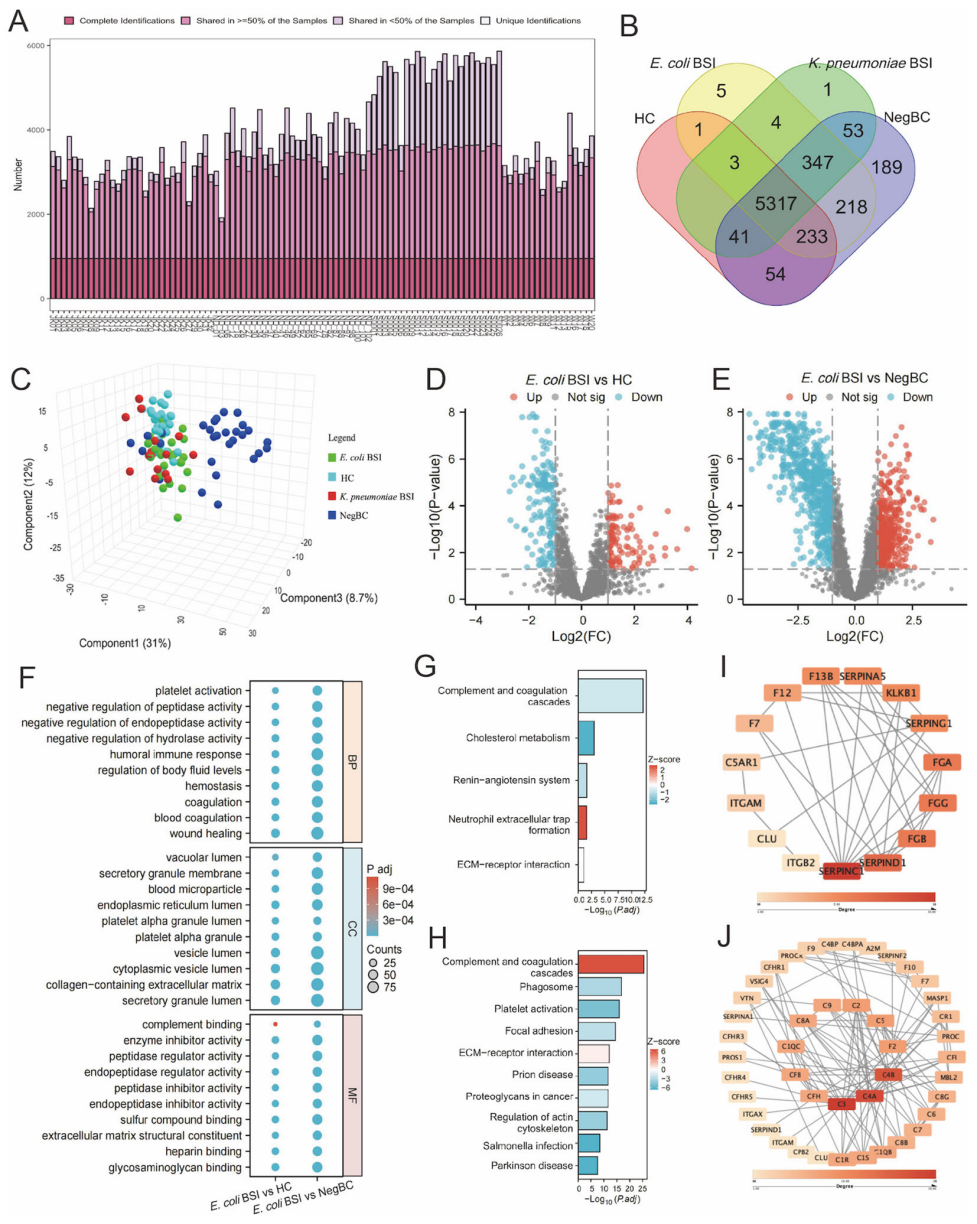
Proteomic profiles of *E. coli* BSI compared with HC and NegBC. **(A)**, Number of identified proteins across samples. **(B)**, Venn diagram showing the distribution of identified proteins among HC, *E. coli* BSI, *K. pneumoniae* BSI, and NegBC. **(C)**, PLS-DA score plot showing the global plasma proteomic profiles. **(D, E)**, Volcano plots showing differentially expressed proteins in *E. coli* BSI versus HC and *E. coli* BSI versus NegBC. **(F)**, GO enrichment analysis of DEPs that remained significant after covariate adjustment, showing representative enriched terms in biological process, cellular component, and molecular function categories. **(G, H)**, KEGG pathway enrichment analysis based on covariate-adjusted DEPs in *E. coli* BSI versus HC and *E. coli* BSI versus NegBC. The Z-score indicates pathway directionality and was calculated as (Up − Down)/√Counts using the GOplot framework. **(I, J)**, Protein-protein interaction networks of covariate-adjusted DEPs involved in the complement and coagulation cascades pathway in *E. coli* BSI versus HC and *E. coli* BSI versus NegBC. Redder colors indicate higher relative expression or enrichment intensity.

In the proteomic analysis, 6,466 proteins were identified in total, and the distribution of identified proteins across samples and groups is shown in **Figures 2A, B**. After quality filtering, 3,737 proteins were retained for downstream proteomic analysis. PLS-DA showed clear separation of global proteomic profiles across groups before covariate adjustment (**Figure 2C**), and PCA including QC samples further supported the overall proteomic data structure (**Supplementary Figure 1E**). Compared with HC and NegBC, *E. coli* BSI exhibited 271 and 1,103 DEPs before covariate adjustment, respectively (**Figures 2D, E**), of which 87 overlapped between the two comparisons (**Supplementary Figures 1F, G**). After covariate adjustment, the retained DEPs were used for biological enrichment analyses. GO enrichment analysis linked these covariate-adjusted DEPs to blood coagulation, humoral immune response, hemostasis, platelet activation, and platelet alpha-granule-related terms (**Figure 2F**). KEGG analysis consistently highlighted the complement and coagulation cascades as a dominant enriched pathway, together with platelet activation and other immune/coagulation-related pathways (**Figures 2G, H**). PPI analysis further showed that coagulation factors, SERPIN family members, and complement components formed a tightly connected network module in the complement and coagulation cascades pathway (**Figures 2I, J**), supporting the presence of a prominent thromboinflammatory response pattern in *E. coli* BSI.

**Figure 3:**
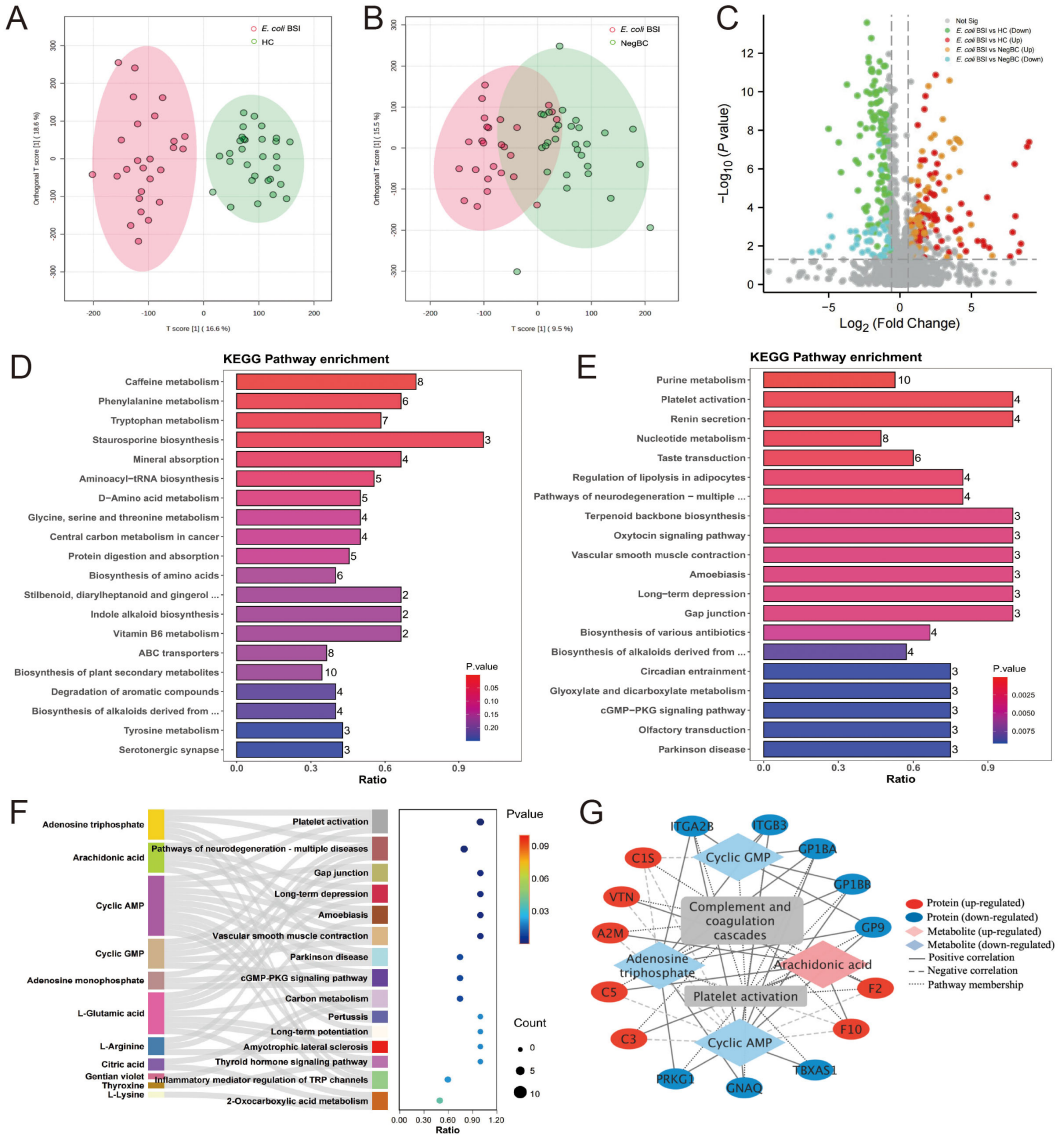
Metabolomic and integrated multi-omics profiles of *E. coli* BSI compared with HC and NegBC. **(A, B)**, OPLS-DA score plots showing metabolomic separation in *E. coli* BSI versus HC and *E. coli* BSI versus NegBC in positive ion mode. **(C)**, Volcano plot showing differentially expressed metabolites in positive ion mode. **(D, E)**, KEGG pathway enrichment analysis based on DEMs that remained significant after covariate adjustment in *E. coli* BSI versus HC and *E. coli* BSI versus NegBC. **(F)**, Sankey diagram showing shared KEGG pathways between DEPs and DEMs that remained significant after covariate adjustment. **(G)**, Focused protein–metabolite network linking the complement and coagulation cascades with platelet activation in *E. coli* BSI versus NegBC. Only protein–metabolite correlations with |Spearman ρ| ≥ 0.55 and FDR < 0.05 are shown.

For the metabolomic dataset, QC correlation matrices and PCA plots in both positive and negative ion modes also supported analytical stability during acquisition (**Supplementary Figures 2A–D**). In the metabolomic analysis, 1,579 compounds were detected across both ionization modes. OPLS-DA suggested distinct metabolic profiles for *E. coli* BSI relative to HC and NegBC before covariate adjustment (**Figures 3A, B**), and the corresponding permutation tests are shown in **Supplementary Figures 3A, B**. Before covariate adjustment, a substantial number of DEMs were identified in *E. coli* BSI versus HC and *E. coli* BSI versus NegBC. In positive ion mode, 73 metabolites were upregulated and 113 were downregulated in *E. coli* BSI versus HC, whereas 59 were upregulated and 36 were downregulated in *E. coli* BSI versus NegBC (**Figure 3C**; **Supplementary Figures 3C, D**). These DEMs were mainly composed of fatty acids and hydroxy acids, amino acids and derivatives, methylated derivatives, and other organic compounds, with a smaller proportion of lipid signaling molecules/oxylipins. After covariate adjustment, the retained DEMs were used for KEGG pathway enrichment analysis. Pathway enrichment showed broader metabolic perturbation in *E. coli* BSI versus NegBC than in *E. coli* BSI versus HC (**Figures 3D, E**), suggesting more extensive metabolic reprogramming relative to culture-negative sepsis than relative to healthy controls. These altered pathways involved lipid metabolism, amino acid metabolism, nucleotide-related metabolism, cGMP-PKG signaling, and platelet-related signaling.

To integrate the proteomic and metabolomic data, we examined the overlap of significantly enriched KEGG pathways based on DEPs and DEMs that remained significant after covariate adjustment. No shared enriched KEGG pathway was observed in *E. coli* BSI versus HC, whereas 15 shared pathways were identified in *E. coli* BSI versus NegBC, including platelet activation, carbon metabolism, the cGMP-PKG signaling pathway, and long-term potentiation (**Figure 3F**). Key metabolites contributing to these pathways included cyclic AMP, arachidonic acid, ATP, cyclic GMP, and L-glutamic acid. Among these shared pathways, selected immune- and inflammation-related pathways were further examined to characterize their pathway-level relationships. Because complement and coagulation cascades was the dominant protein-level KEGG pathway and platelet activation was identified as a shared proteomic–metabolomic pathway, we further inspected their connection at the protein-pathway level. Coagulation factor II (F2) was annotated to both the complement and coagulation cascades pathway and the platelet activation pathway, highlighting F2 as a bridging protein between complement/coagulation activation and platelet activation (**Supplementary Figure 3E**). A focused protein–metabolite network of the complement and coagulation cascades and platelet activation pathways revealed complex coordinated perturbations involving coagulation, platelet signaling, and related metabolic regulation (**Figure 3G**). At the protein-level pathway comparison across both control contrasts, *E. coli* BSI showed recurrent enrichment of complement and coagulation cascades and cholesterol metabolism, with additional involvement of Fc gamma receptor-mediated phagocytosis, neutrophil extracellular trap formation, ECM-receptor interaction, and focal adhesion. Overall, these findings suggest that *E. coli* BSI is characterized by coordinated thromboinflammatory and platelet-centered immunometabolic perturbation.

### 3.3 Plasma multi-omics characteristics of *K. pneumoniae* BSI: coupling of coagulation-immune signaling and lipid metabolism

**Figure 4:**
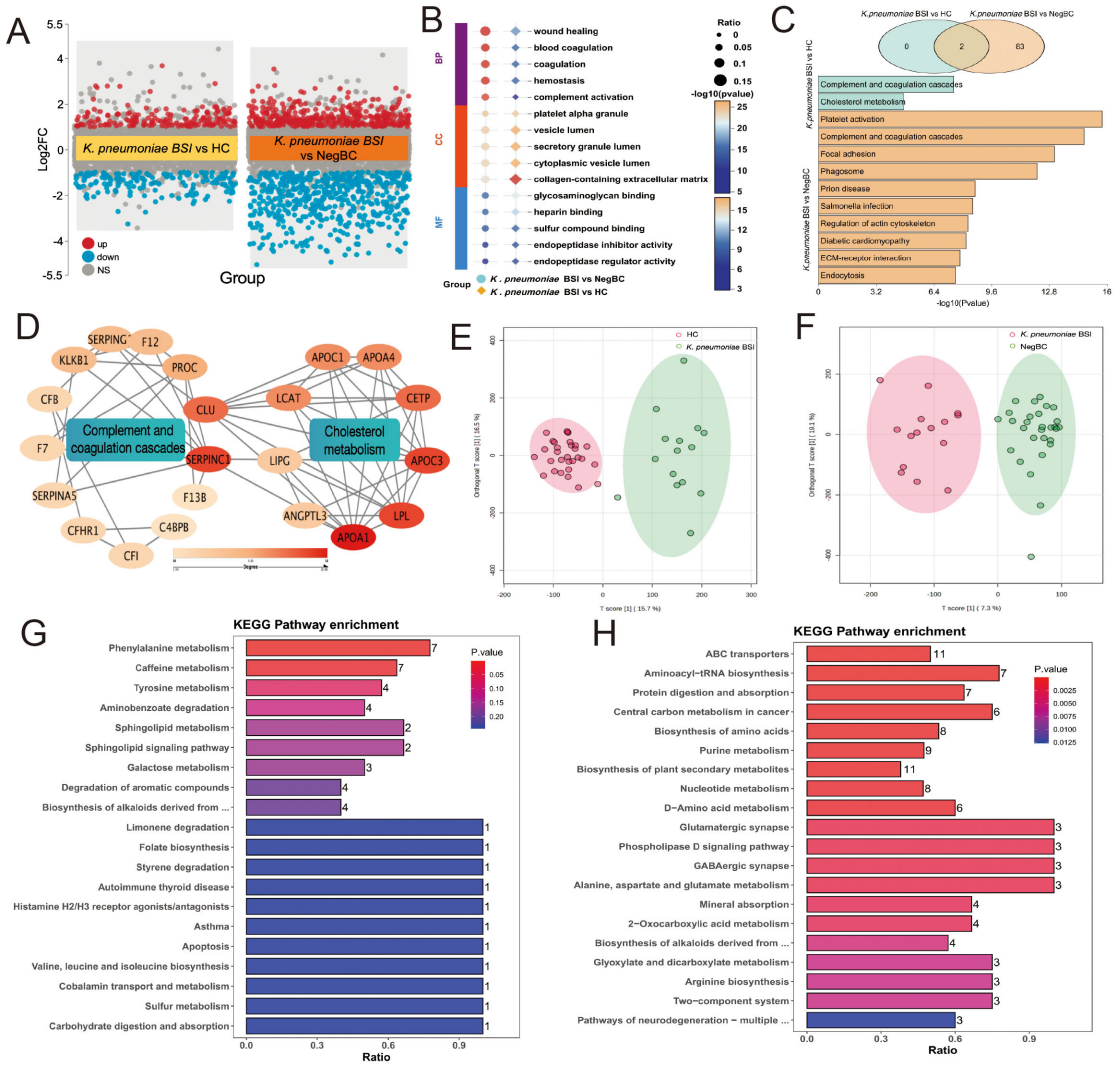
Proteomic and metabolomic profiles of *K. pneumoniae* BSI compared with HC and NegBC. **(A)**, Volcano plots showing differentially expressed proteins in *K. pneumoniae* BSI versus HC and *K. pneumoniae* BSI versus NegBC. **(B)**, GO enrichment analysis of DEPs that remained significant after covariate adjustment, showing representative enriched terms shared across comparisons. **(C)**, KEGG pathway enrichment analysis showing the top 10 pathways ranked by *P* value in *K. pneumoniae* BSI versus HC and *K. pneumoniae* BSI versus NegBC. Two pathways were shared between the two comparisons. **(D)**, Protein-protein interaction network of covariate-adjusted DEPs involved in complement/coagulation and cholesterol metabolism in *K. pneumoniae* BSI. **(E, F)**, OPLS-DA score plots showing metabolomic separation in positive ion mode. **(G, H)**, KEGG pathway enrichment analysis based on DEMs that remained significant after covariate adjustment in *K. pneumoniae* BSI versus HC and *K. pneumoniae* BSI versus NegBC.

Proteomic analysis showed that *K. pneumoniae* BSI differed from HC and NegBC by 320 and 905 proteins before covariate adjustment, respectively (**Figure 4A**). Across these two comparisons, a shared response set was identified (**Supplementary Figures 4A, B**). After covariate adjustment, the retained DEPs were used for biological enrichment analyses. GO enrichment analysis showed that these DEPs were mainly related to coagulation/hemostasis, humoral immune response, complement activation, and platelet alpha-granule biology (**Figure 4B**). KEGG pathway enrichment showed the top 10 pathways ranked by *P* value in *K. pneumoniae* BSI versus HC and *K. pneumoniae* BSI versus NegBC, with two pathways shared between the two comparisons (**Figure 4C**). These shared signals mainly involved complement and coagulation cascades and cholesterol metabolism. PPI analysis further showed that proteins involved in complement/coagulation and cholesterol metabolism formed a connected network module, supporting a relatively focused axis involving coagulation-immune activation and lipid metabolic remodeling in *K. pneumoniae* BSI (**Figure 4D**; **Supplementary Figure 4C**).

Metabolomically, *K. pneumoniae* BSI also separated from HC and NegBC before covariate adjustment in positive ion mode (**Figures 4E, F**), and the corresponding OPLS-DA permutation tests are shown in **Supplementary Figures 4E, F**. A substantial number of DEMs were identified in *K. pneumoniae* BSI compared with HC and NegBC before covariate adjustment. Most of these metabolites belonged to fatty acids and hydroxy acids, amino acids and derivatives, methylated derivatives, and other organic compounds, with very few lipid signaling molecules/oxylipins. These results suggest that, similar to *E. coli* BSI, *K. pneumoniae* BSI was also accompanied by fatty acid-related metabolic perturbation. After covariate adjustment, the retained DEMs were used for KEGG pathway enrichment analysis. Pathway enrichment revealed only two significant pathways in *K. pneumoniae* BSI versus HC, namely caffeine metabolism and phenylalanine metabolism, whereas 48 pathways were enriched in *K. pneumoniae* BSI versus NegBC. These pathways involved ABC transporters, amino acid metabolism and biosynthesis, purine/pyrimidine metabolism, carbon metabolism, and related metabolic processes (**Figures 4G,H**). Although *K. pneumoniae* BSI versus NegBC also showed protein-level changes related to complement/coagulation and platelet-associated biology, these signals did not converge on a platelet-centered shared proteomic–metabolomic network to the same extent as observed for *E. coli* BSI versus NegBC. Overall, these findings suggest that *K. pneumoniae* BSI is characterized by coagulation-immune activation coupled with lipid and broader metabolic remodeling.

### 3.4 Exploratory comparison between *E. coli* BSI and *K. pneumoniae* BSI

**Figure 5:**
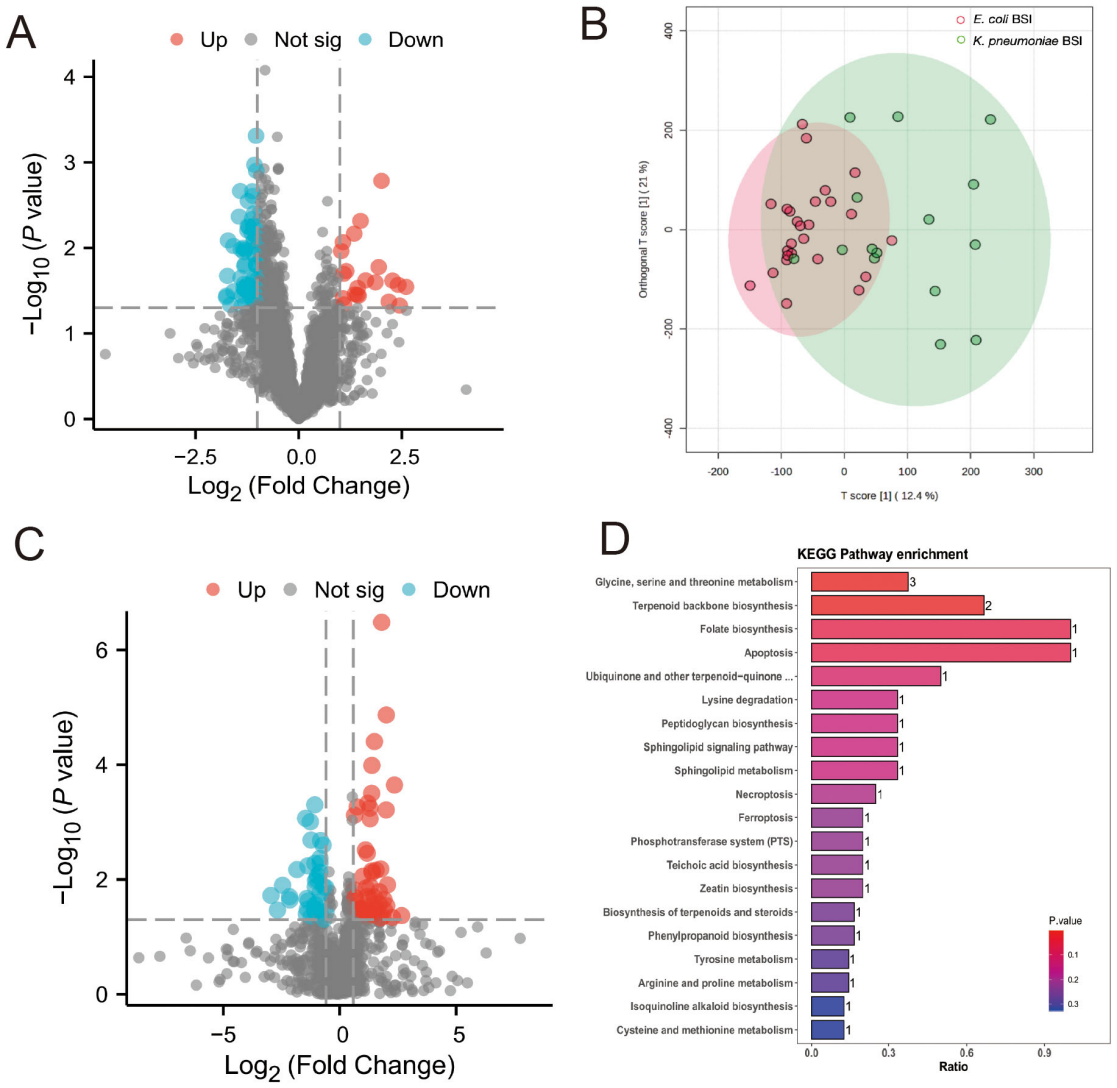
Exploratory multi-omics comparison between *E. coli* BSI and *K. pneumoniae* BSI. **(A)**, Volcano plot showing protein-level differences before covariate adjustment. **(B)**, OPLS-DA score plot showing global metabolomic profiles before covariate adjustment. **(C)**, Volcano plot showing differentially expressed metabolites between *E. coli* BSI and *K. pneumoniae* BSI. **(D)**, KEGG pathway enrichment analysis based on DEMs that remained significant after covariate adjustment; the top 20 pathways are shown.

To directly compare pathogen-associated host-response patterns, we performed a head-to-head multi-omics comparison between *E. coli* BSI and *K. pneumoniae* BSI. Before covariate adjustment, the proteomic comparison showed differential protein-expression patterns between the two pathogen groups (**Figure 5A**). However, after adjustment for baseline clinical covariates, no proteins met the predefined criteria for differential expression between *E. coli* BSI and *K. pneumoniae* BSI. This finding indicates that the robust protein-level separation between the two pathogen groups was limited after accounting for baseline clinical heterogeneity.

At the metabolomic level, the global separation between *E. coli* BSI and *K. pneumoniae* BSI was modest before covariate adjustment (**Figure 5B**). Nevertheless, a subset of metabolites remained significantly different after covariate adjustment. KEGG pathway enrichment analysis of these retained DEMs identified four significantly altered pathways: glycine, serine and threonine metabolism; terpenoid backbone biosynthesis; apoptosis; and folate biosynthesis (**Figures 5C, D**). These pathways suggest that *E. coli* BSI and *K. pneumoniae* BSI may differ in selected amino acid metabolism, isoprenoid-related biosynthetic processes, cell death-related regulation, and folate-associated metabolism.

### 3.5 *E. coli* BSI diagnostic model and exploratory *K. pneumoniae* BSI model

**Figure 6:**
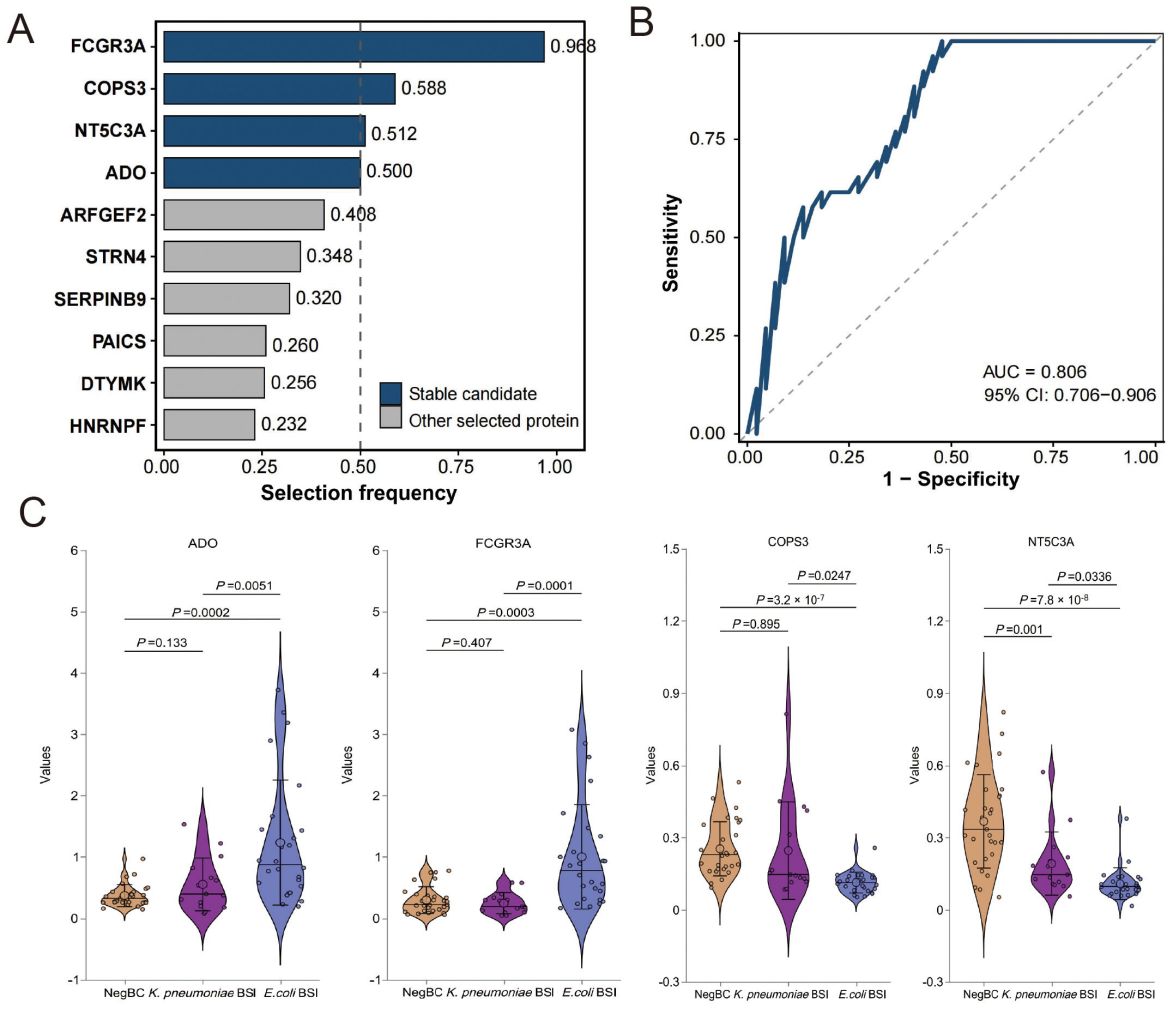
Leakage-free plasma proteome-based diagnostic model for *E. coli* BSI. **(A)** Selection frequencies of candidate proteins in the leakage-free internal validation framework. **(B)** ROC curve based on subject-level averaged held-out predictions for distinguishing *E. coli* BSI from non-*E. coli* sepsis. **(C)** Quantitative abundance comparison of the stable candidate proteins retained in the *E. coli* BSI diagnostic model.

To evaluate pathogen-informative diagnostic signatures in a clinically relevant sepsis-discrimination setting, we constructed a plasma proteome-based model to distinguish *E. coli* BSI from non-*E. coli* sepsis. HC samples were excluded from model construction, and the non-*E. coli* sepsis group comprised *K. pneumoniae* BSI and NegBC. Using the prespecified leakage-free internal validation framework, four relatively stable candidate proteins were identified for the *E. coli* BSI model: FCGR3A, COPS3, NT5C3A, and ADO (**Figure 6A**; **Supplementary Table 1**). Based on subject-level averaged held-out predictions, the model achieved an AUC of 0.806 (95% CI: 0.706–0.906) for distinguishing *E. coli* BSI from non-*E. coli* sepsis (**Figure 6B**; **Supplementary Table 2**). The four retained candidate proteins displayed group-specific differences in quantitative abundance (**Figure 6C**). These results suggest that plasma proteomic features may provide exploratory discriminatory information for *E. coli* BSI within the sepsis population. However, because the model was evaluated only by internal cross-validation in a single-center cohort, its diagnostic performance should be considered preliminary and requires external validation in larger independent cohorts. Because of the limited sample size of the *K. pneumoniae* BSI group, the *K. pneumoniae* BSI diagnostic model was reported only as an exploratory **Supplementary Material Analysis**.

## 4 Discussion

This study used plasma proteomic and metabolomic profiling to characterize host-response patterns in *E. coli* BSI and *K. pneumoniae* BSI. Overall, both Gram-negative BSI groups showed prominent coagulation-immune activation, whereas *E. coli* BSI showed a more evident platelet-centered immunometabolic network and *K. pneumoniae* BSI showed coagulation-immune activation coupled with lipid and broader metabolic remodeling. A plasma proteome-based model further suggested exploratory diagnostic value for distinguishing *E. coli* BSI from non-*E. coli* sepsis.

Sepsis is increasingly recognized as a heterogeneous syndrome involving intertwined immune, metabolic, vascular, and coagulation responses rather than a uniform inflammatory state. Recent transcriptomic and multi-omics studies have identified reproducible sepsis endotypes and molecular subgroups characterized by differences in inflammation, immune suppression, coagulation, metabolism, and treatment response [Chenoweth et al., 2024; Zhang et al., 2025]. Recent transcriptomic and multi-omics studies have emphasized that sepsis-related molecular phenotypes often reflect coordinated immune, vascular, coagulation, and metabolic programs. In the present cohort, the persistence of coagulation/complement, humoral immune, platelet-related, and metabolic signals after covariate adjustment is compatible with this broader view, while the separate analyses of *E. coli* BSI and *K. pneumoniae* BSI suggest that this shared host-response background may be accompanied by pathogen-associated differences in platelet-related and metabolic remodeling.

Complement and coagulation cascades were repeatedly implicated in both *E. coli* BSI and *K. pneumoniae* BSI when each pathogen group was compared with HC or NegBC. This observation is consistent with current concepts of sepsis-associated thromboinflammation, in which pathogen-triggered innate immune activation intersects with endothelial dysfunction, complement activation, thrombin generation, platelet activation, and leukocyte recruitment [Iba and Levy, 2020; Jiang et al., 2024; Giamarellos et al., 2024; Kappelmayer et al., 2024; Aklilu et al., 2025]. In the present cohort, these pathway-level findings support thromboinflammation as a shared host-response feature of *E. coli* BSI and *K. pneumoniae* BSI. However, this conclusion should be interpreted within the boundaries of the study design. Because Gram-positive and viral sepsis were not included, our data cannot determine whether this pattern is specific to Gram-negative BSI or represents a broader feature across different sepsis etiologies.

Beyond this shared thromboinflammatory background, *E. coli* BSI showed a more evident platelet-centered immunometabolic pattern, particularly in comparison with NegBC. Platelets are now recognized not only as hemostatic effectors but also as immune mediators that interact with complement, coagulation factors, leukocytes, and endothelial cells during infection and sepsis [Yang et al., 2025]. In our integrated analysis, platelet activation, cGMP-PKG signaling, arachidonic acid, cyclic AMP, cyclic GMP, and ATP were involved in the *E. coli* BSI-associated proteomic–metabolomic network. The focused protein–metabolite network linking complement/coagulation cascades and platelet activation further suggested coordinated perturbations involving coagulation, platelet signaling, and related metabolic regulation. These findings are consistent with recent pathogen-oriented host-response studies showing that bloodstream infection can induce both conserved inflammatory programs and pathogen-associated molecular differences [Thaden et al., 2024; Butler et al., 2025]. However, broader pathogen classes, such as Gram-negative versus Gram-positive bloodstream infections, may induce more clearly distinguishable host transcriptional patterns, whereas host-response differences between closely related Gram-negative pathogens such as *E. coli* and *K. pneumoniae* are more limited and require cautious interpretation [Thaden et al., 2024].

*K. pneumoniae* BSI also showed coagulation-immune activation, but its multi-omics profile appeared more closely coupled to lipid and broader metabolic remodeling. The adjusted proteomic analyses continued to implicate coagulation/hemostasis, humoral immune response, complement activation, and cholesterol metabolism, while the adjusted metabolomic analysis showed broader pathway enrichment in *K. pneumoniae* BSI versus NegBC. This pattern is compatible with the biological characteristics of *K. pneumoniae*, including capsule-mediated immune evasion, siderophore-mediated iron acquisition, and host metabolic adaptation [Wong Fok Lung et al., 2022; Monteiro et al., 2024; Bai et al., 2024]. Experimental and clinical studies also suggest that *K. pneumoniae* infection can reshape host metabolism and stress responses during infection [Wong Fok Lung et al., 2022]. In addition, lipid and lipoprotein remodeling is increasingly recognized as an important component of sepsis immunometabolism, with potential implications for inflammation, microbial defense, and disease severity [Palmowski et al., 2025; Augustin et al., 2024]. Therefore, the lipid and metabolic remodeling observed in *K. pneumoniae* BSI may reflect both general sepsis immunometabolic changes and pathogen-associated host metabolic adaptation.

In the direct comparison between *E. coli* BSI and *K. pneumoniae* BSI, no proteins met the predefined criteria for differential expression after covariate adjustment. Therefore, we did not further perform protein-level GO, KEGG, or PPI analyses to support strong pathogen-specific mechanisms in this direct comparison. Instead, selected metabolomic differences persisted after covariate adjustment, involving glycine, serine and threonine metabolism, terpenoid backbone biosynthesis, apoptosis, and folate biosynthesis. These pathways suggest that metabolic rather than proteomic differences may capture some residual pathogen-associated divergence between the two Gram-negative BSI groups. Given the sample size and modest direct separation, these findings should be considered exploratory and hypothesis-generating.

The *E. coli* BSI diagnostic model was reconstructed using a leakage-free internal validation framework and excluded HC samples to better reflect a clinically relevant sepsis-discrimination setting. The final model retained four relatively stable candidate proteins: FCGR3A, COPS3, NT5C3A, and ADO. FCGR3A encodes Fc gamma receptor IIIa/CD16a, a receptor involved in antibody-dependent immune effector functions and the interface between humoral and innate immunity [Coënon and Villalba, 2022; Frampton et al., 2024]. Its retention in the model is consistent with the prominent humoral immune and complement-related signatures observed in *E. coli* BSI. COPS3 is a component of the COP9 signalosome, which has been implicated in the regulation of NF-κB signaling, innate immunity, and inflammatory responses [Schweitzer and Naumann, 2010; Guo et al., 2024]. NT5C3A is a pyrimidine 5′-nucleotidase reported to negatively regulate interferon and cytokine signaling [Al-Haj et al., 2018]. ADO, also known as 2-aminoethanethiol dioxygenase, is involved in thiol metabolism and oxygen sensing [Patel et al., 2024]. Together, these proteins may reflect an *E. coli* BSI-associated host state involving Fc receptor-mediated immune activation, inflammatory regulation, nucleotide metabolism, and oxygen/thiol metabolic adaptation. However, their pathogen specificity has not been independently validated, and they should currently be regarded as candidate biomarkers rather than established diagnostic markers.

Because of the limited sample size of the *K. pneumoniae* BSI group, the *K. pneumoniae* BSI diagnostic model was retained only as an exploratory supplementary analysis and was not emphasized as a main diagnostic finding. Moreover, LC-MS/MS-based multi-omics should be viewed as a discovery platform rather than a ready-to-use rapid diagnostic test. Although blood cultures have delayed turnaround time, untargeted multi-omics workflows are also time-consuming and costly. Future translation will require independent validation of the candidate proteins and conversion into faster targeted assays, such as PRM/MRM, ELISA, or multiplex immunoassays [Parker and Borchers, 2014; Babaei et al., 2024; Wenk et al., 2024].

Several limitations should be acknowledged. First, this was a single-center study with a limited sample size, especially for the *K. pneumoniae* BSI group, which reduces statistical power and limits the stability of subgroup analyses and diagnostic modeling. Second, although we adjusted for key clinical covariates including sex, lactate, and APACHE II score, residual confounding cannot be fully excluded. Third, the study included only *E. coli* BSI, *K. pneumoniae* BSI, NegBC, and HC; therefore, the findings cannot be generalized to Gram-positive or viral sepsis. Fourth, the diagnostic model was evaluated only by internal cross-validation and requires external validation in larger multicenter cohorts. Finally, although all plasma samples were collected before antimicrobial administration, the NegBC group remains clinically heterogeneous and may include patients with different infection sources, pathogen burdens, or nonbacteremic inflammatory states.

## 5 Conclusion

In summary, plasma multi-omics suggested that *E. coli* BSI and *K. pneumoniae* BSI share thromboinflammatory features involving complement/coagulation and immune activation, while also showing partially different platelet-associated and metabolic patterns. *E. coli* BSI appeared to be associated with a platelet-centered immunometabolic network, whereas *K. pneumoniae* BSI was more closely linked to lipid and broader metabolic remodeling. These findings should be interpreted cautiously given the sample size and need for external validation, but they provide a pathogen-oriented framework for future validation of targeted biomarkers in Gram-negative BSI.

## Data Availability

The datasets presented in this study can be found in online repositories. The names of the repository/repositories and accession number(s) can be found below: http://www.proteomexchange.org/, PXD077707 http://www.proteomexchange.org/, PXD077865.
