# Supplementary material for: Plasma multi-omics reveals pathogen-associated mechanisms and diagnostic signatures in Escherichia coli and Klebsiella pneumoniae bloodstream infections
Source: Front Cell Infect Microbiol. 2026 Jul 9;16:1873717. doi: 10.3389/fcimb.2026.1873717 (PMC13391253; doi:10.3389/fcimb.2026.1873717)
Supplement: Supplementary file 3 [file Supplementaryfile1.docx]

Supplementary Material

# Supplementary Data

**1.1 Methods**

**1.1.1 Proteins Sequencing and Data Preprocessing**

**Sample preparation**

All plasma samples were processed as described in a previous study [Wang et al., 2023]. Briefly, the Qinglian Low-Abundance Protein Enrichment Magnetic Bead Kit (Beijing Qinglian Biotech Co., Ltd., DMB3000-96-E) was used. Specifically, 50 μL of each sample was mixed with the diluent (DMB-DB-2) and then added to DMB beads that had been pre-incubated with the same diluent. The mixture was incubated with shaking at 1000 rpm and 37 °C for 30 minutes. After magnetic removal of the supernatant, the beads were washed three times with the diluent to obtain magnetic beads containing low-abundance proteins. Subsequently, the beads were resuspended in a lysis buffer (50 mM TEAB, 10 mM TCEP, 50 mM IAA). The mixture was then subjected to reduction and alkylation at 95 °C for 10 min, followed by incubation with 80% acetonitrile for 20 min. After that, the solvent was exchanged with 50 mM ammonium bicarbonate solution, and the mixture was digested with trypsin at 37 °C for 4 h. The digestion was stopped by adding 0.1% TFA buffer. The digested peptides were quantified using a NanoDrop, and then prepared for LC-MS/MS analysis.

**LC-MS/MS Analysis**

Prepare mobile phase A (100% water, 0.1% formic acid) and mobile phase B (80% acetonitrile, 0.1% formic acid). Dissolve 10 µL of mobile phase A in the lyophilized powder, and centrifuge at 14,000 g for 20 minutes at 4 ℃. 400 ng of peptide were loaded on a 25 cm column (100 μm inner diameter, packed using ReproSil-Pur C18-AQ 1.5- µm silica beads;  QL-HPLC-100*15；Beijing Qinglian Biotech Co.,Ltd, Beijing, China). Peptides were separated using a gradient from 3.5 to 32% B in 17 min , then 32% to 95% B in 1 min and holding it at 95% for 2 min, then 95% to 1 % B in 2 min. Use the timsTOF_HT mass spectrometer (Bruker Corporation, USA) with the Captive Spray ion source. For DIA (Data-Independent Acquisition), the Mass Width was set to 10 Da. The Mass range covered from 368.5 to 1,098.5 and was separated into 73 acquisition windows. In the TIMS tunnel, set the accumulation time to 50 ms. The capillary voltage is set to 1.5 kV, with a mobility range of 0.7 to 1.3 cm²/(V). The total cycle time is 1.23 seconds.

**1.1.2 The identification and quantitation of protein**

RAW files were analyzed using the Spectronaut software (Biognosys, version 15.7.220308.50606). All searches were performed against the human UniProt reference proteome of canonical and isoform sequences. Searches used carbamidomethylation as fixed modification and acetylation of the protein N-terminus, oxidation of methionines as variable modifications. Default settings were used for other parameters. In brief, a trypsin/P proteolytic cleavage rule was used, permitting a maximum of two miscleavages and a peptide length of 7–52 amino acids. Protein intensities were normalized using the “Local Normalization” algorithm in Spectronaut based on a local regression model. Spectral library generation stipulated a minimum of three fragments per peptide, and maximally, the six best fragments were included. A protein and precursor FDR of 1% were used and protein quantities were reported in samples only if the protein passed the filter.

**1.1.3 Metabolomics Sequencing and Data Preprocessing**

**Sample Processing and Mass Spectrometry**

Identification. Samples were placed on dry ice, and 100 µL of the sample was combined with 400 µL of extraction solvent (methanol: acetonitrile = 3:1, pre-cooled at -40 °C). The extraction solvent contained 2,6-dichlorophenylalanine (internal standard, concentration of 100 ng/mL). The mixture was vortexed for 5 minutes and left to stand at 4 °C for 2 hours. The samples were then centrifuged at 12,000 rpm for 15 minutes at 4 °C. An equal volume was taken and vacuum concentrated to dryness. Subsequently, 100 µL of a 50% methanol aqueous solution (methanol: water = 1:1, v/v) was added for re-dissolution, followed by vortexing for 3 minutes (at 4 °C, 2000 rpm) and centrifugation for 15 minutes (12,000 rpm, 4 °C). The supernatant was collected for injection analysis. Prior to injection, QC samples were tested to monitor the instrument's status and balance the chromatography-mass spectrometry system. QC samples were then inserted during the sample analysis to evaluate the platform's stability throughout the experiment and for subsequent data quality control analysis.

**Mass Spectrometry Identification**

Non-targeted metabolomics is based on liquid chromatography-tandem mass spectrometry (LC-MS/MS) technology, employing both positive (pos) and negative (neg) ion data acquisition modes for comprehensive and high-throughput detection and analysis of unknown metabolites in samples. After sample pretreatment and metabolite extraction, chromatography is first utilized to separate the metabolites, followed by detection using mass spectrometry. The raw data (.raw) files are processed using Compound Discoverer 3.3 software for peak recognition, peak alignment, peak extraction, and retention time correction, with the following parameter settings: Alignment Model: Adaptive curve; Maximum Shift: 0.5 min; Mass Tolerance: 10 ppm; Intensity Tolerance: 30%; S/N Threshold: 1.5. Missing values are filled using linear regression methods, and samples are normalized to the median peak area of all samples. Compounds are identified using an automated multi-database and spectral library search tool (including mzCloud, Chemspider, Human Metabolome Database (HMDB,https://hmdb.ca/metabolites), and Kyoto Encyclopedia of Genes and Genomes (KEGG,https://www.genome.jp/kegg/pathway. html) and local database search tools (such as the mzVault spectral library) based on secondary mass spectrometry information, with the following parameter settings: Mass tolerance: 10ppm; Match Factor Threshold: 10.

# 1.1.4 Quality control assessment

# Quality control (QC) procedures were performed to evaluate analytical stability and reproducibility of the LC-MS/MS-based proteomic and metabolomic datasets. For proteomic analysis, HeLa cell lysate digest samples were used as external QC standards to monitor mass spectrometry performance. In addition, pooled plasma QC samples prepared from equal aliquots of study samples (QC_H) and commercial plasma standard reference materials (QC_S) were used to assess the reproducibility of the plasma proteomic workflow. All proteomic QC samples were processed using the same data-processing and normalization workflow as the study samples. Pearson correlation coefficients were calculated based on commonly quantified proteins across QC runs.

# For metabolomic analysis, QC samples were inserted throughout the analytical sequence in both positive and negative ion modes. QC performance was assessed using pairwise correlation analysis and principal component analysis (PCA). The correlation consistency and clustering pattern of QC samples were used to evaluate analytical reproducibility and potential technical drift during acquisition.

# All plasma samples in this study were analyzed within the same analytical batch under identical LC-MS/MS conditions. Therefore, no independent analytical batches were introduced, and conventional inter-batch correction was not applicable. Instead, potential technical variation was assessed using the QC-based correlation matrices and PCA plots described above.

**Reference**

Wang J, Xie W, Sun L, Li J, Wu S, Li R, et al. (2023) Establishment and clinical application evaluations of a deep mining strategy of plasma proteomics based on nanomaterial protein coronas. Anal Chim Acta., 22;1275:341569. doi: 10.1016/j.aca.2023.341569.

# 2 Supplementary Figures and Tables

## 2.1 Supplementary Figures


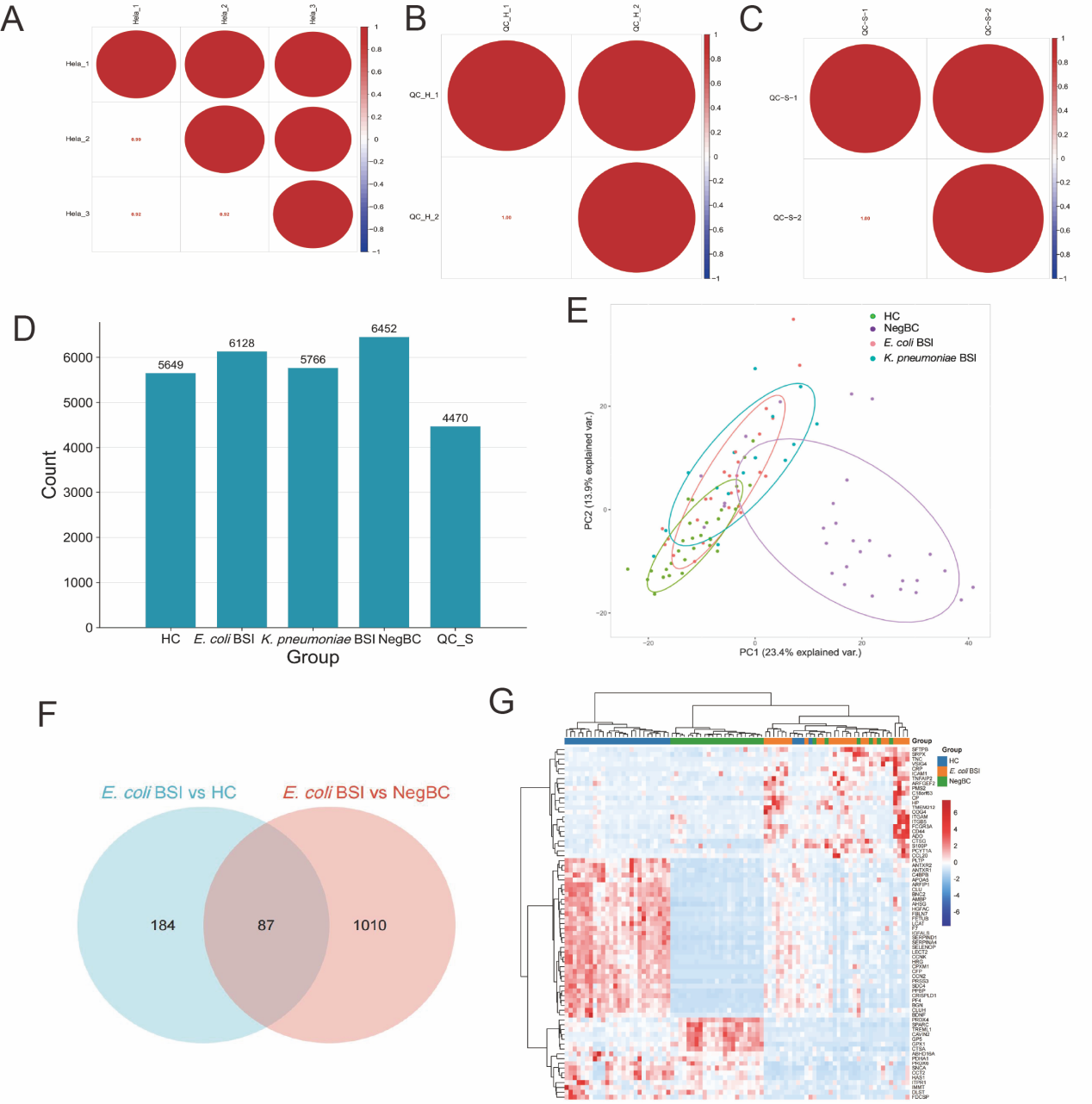


**Figure S1.** Proteomic quality control and auxiliary proteomic overview. **A**, Correlation matrix of HeLa external QC samples in the proteomic analysis. **B**, Correlation matrix of pooled plasma QC samples prepared from study samples. **C**, Correlation matrix of commercial plasma standard QC samples. **D**, Number of identified proteins across HC, *E. coli* BSI, *K. pneumoniae* BSI, NegBC, and QC samples. **E**, PCA plot of global proteomic profiles before covariate adjustment, including study samples and QC samples. **F**, Venn diagram showing the overlap of DEPs before covariate adjustment between *E. coli* BSI versus HC and *E. coli* BSI versus NegBC. **G**, Clustered heatmap of common DEPs before covariate adjustment shared by *E. coli* BSI versus HC and *E. coli* BSI versus NegBC.


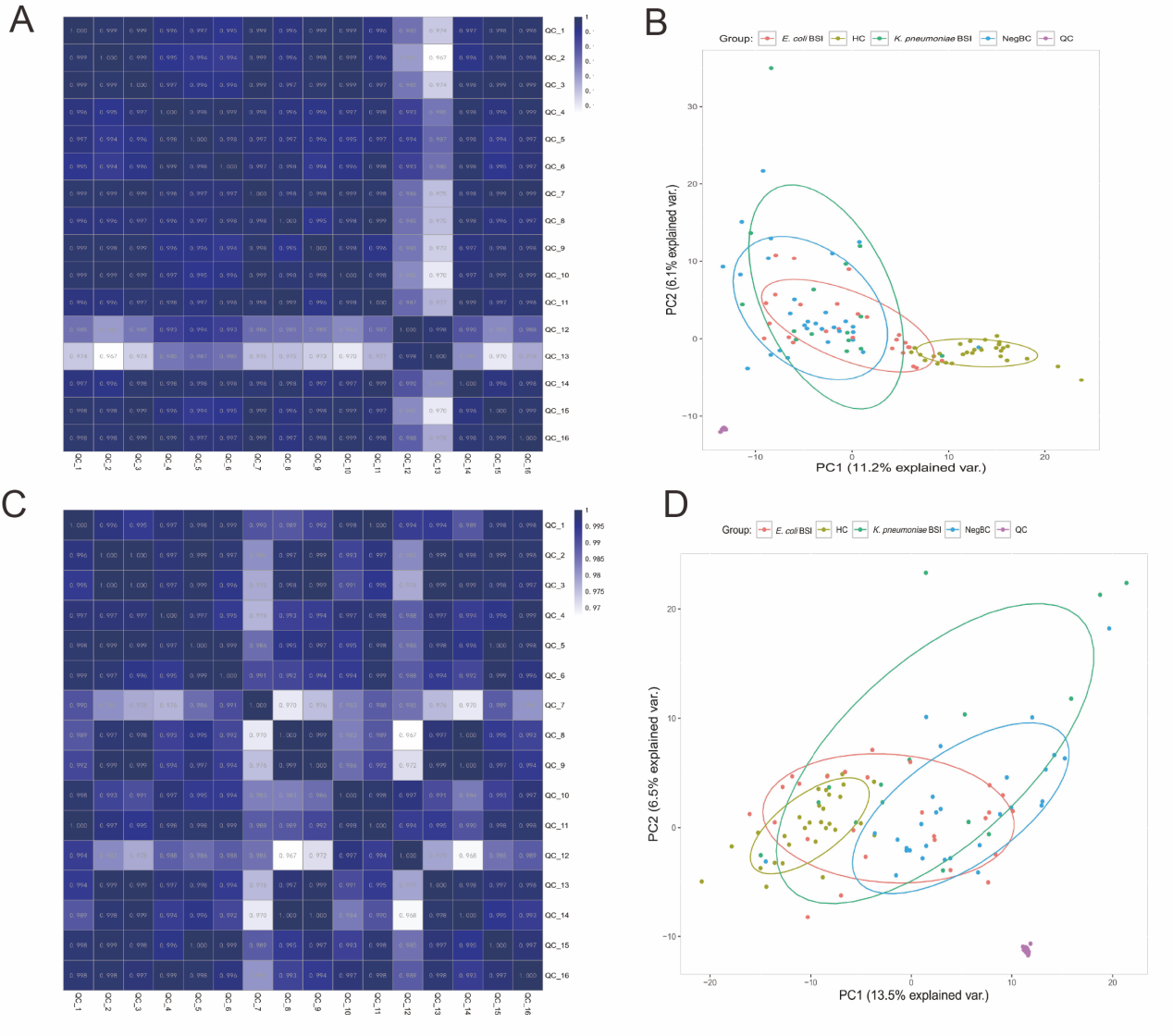


**Figure S2. A**, Correlation matrix of metabolomic QC samples in positive ion mode. **B**, PCA plot of metabolomic samples and QC samples in positive ion mode. **C**, Correlation matrix of metabolomic QC samples in negative ion mode. **D**, PCA plot of metabolomic samples and QC samples in negative ion mode.


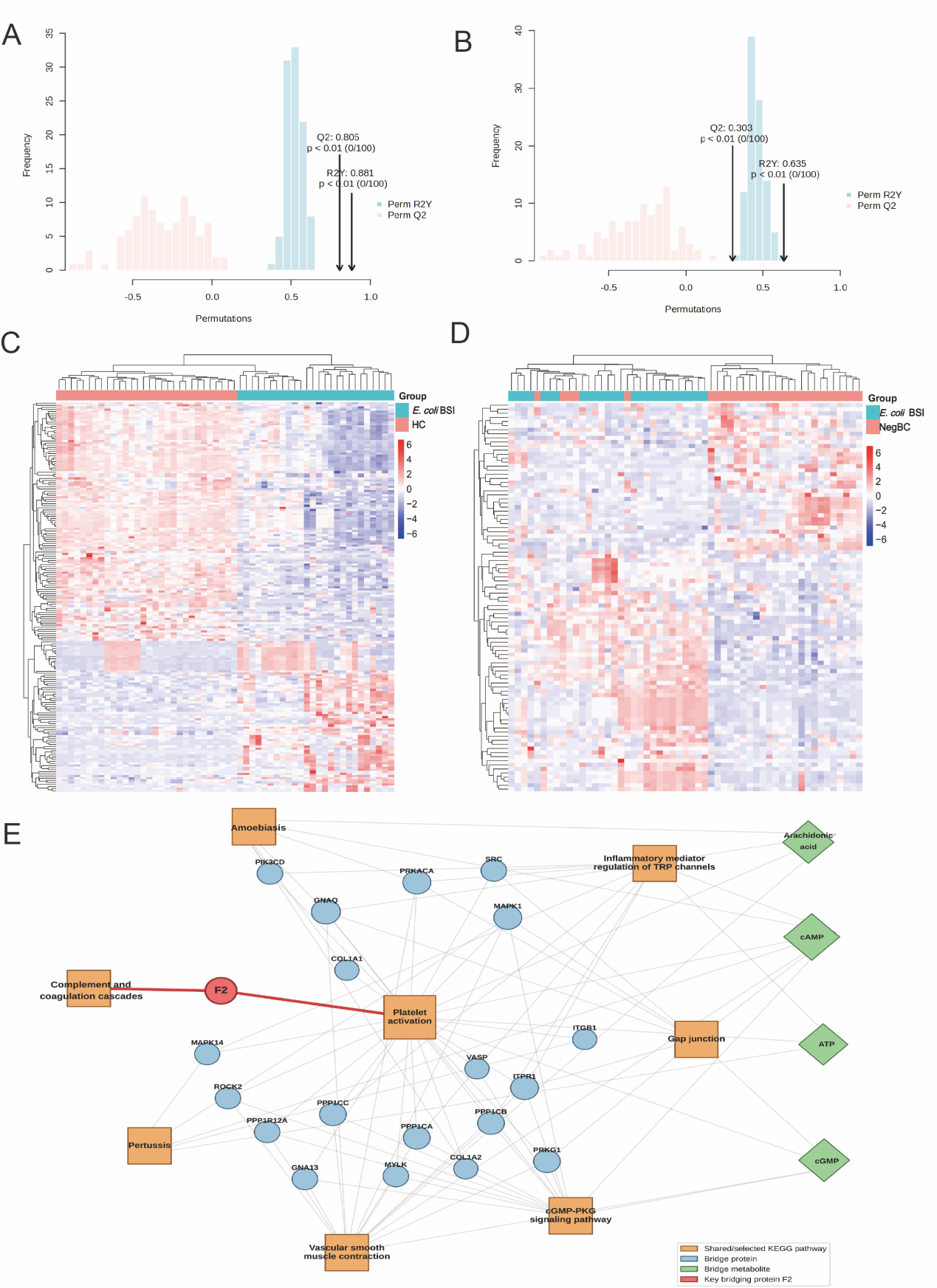


**Figure S3**. **A,** The POS mode of the *E. coli* BSI and HC groups was analyzed using OPLS-DA permutation testing, yielding R²Y = 0.881 and Q² = 0.805. **B,** The POS mode of the *E. coli* BSI and NegBC groups was also analyzed using OPLS-DA permutation testing, resulting in R²Y = 0.635 and Q² = 0.303. **C,** Heatmap of DEMs under the POS mode for the *E. coli* BSI and HC groups. **D,** Heatmap of DEMs under the POS mode for the *E. coli* BSI and NegBC groups. **E,** Core protein–metabolite interaction network of the immune- and inflammation-related pathways in the *E. coli* BSI vs NegBC comparison, Core proteins: occurrence in >3 shared pathways; Core metabolites: occurrence in >2 shared pathways.


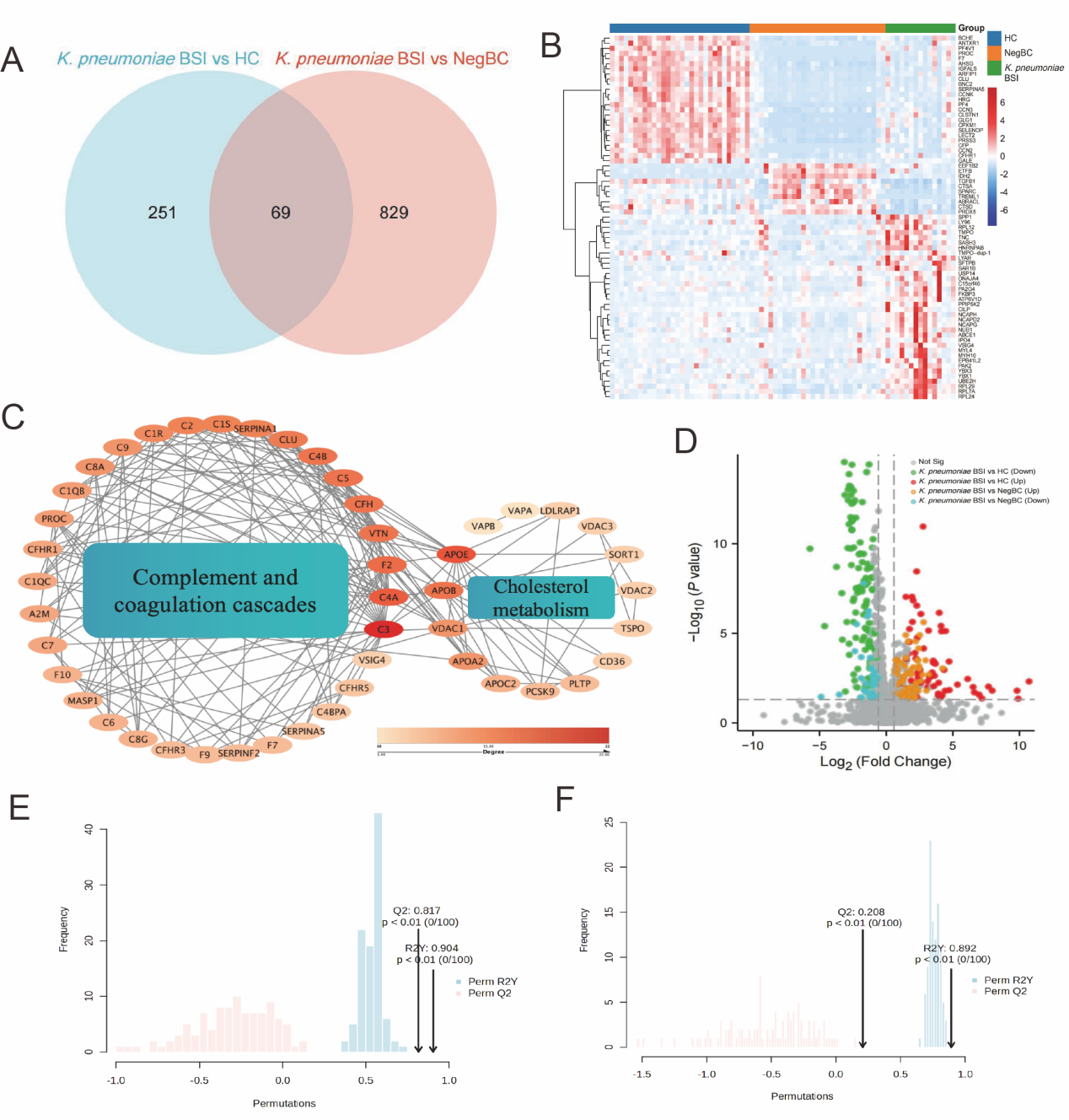


**Figure S4. A,** The Venn diagram of the intersection of DEPs between the *K. pneumoniae* BSI, HC, and NegBC groups; **B,** The heatmap of the 69 common DEPs shared by the *K. pneumoniae* BSI, HC, and NegBC groups; **C,** The protein-protein interaction (PPI) relationships for the common pathways of Complement and coagulation cascades, as well as Cholesterol metabolism, between the *K. pneumoniae* BSI and NegBC groups. **D,** The volcano plot comparing the *K. pneumoniae* BSI, HC, and NegBC groups under the POS mode; **E-F,** The POS mode of the *K. pneumoniae* BSI and HC groups was analyzed using OPLS-DA permutation testing, yielding R2Y = 0.904 and Q2 = 0.817; while the POS mode of the *K. pneumoniae* BSI and NegBC groups was analyzed using OPLS-DA permutation testing, resulting in R2Y = 0.892 and Q2 = 0.208.
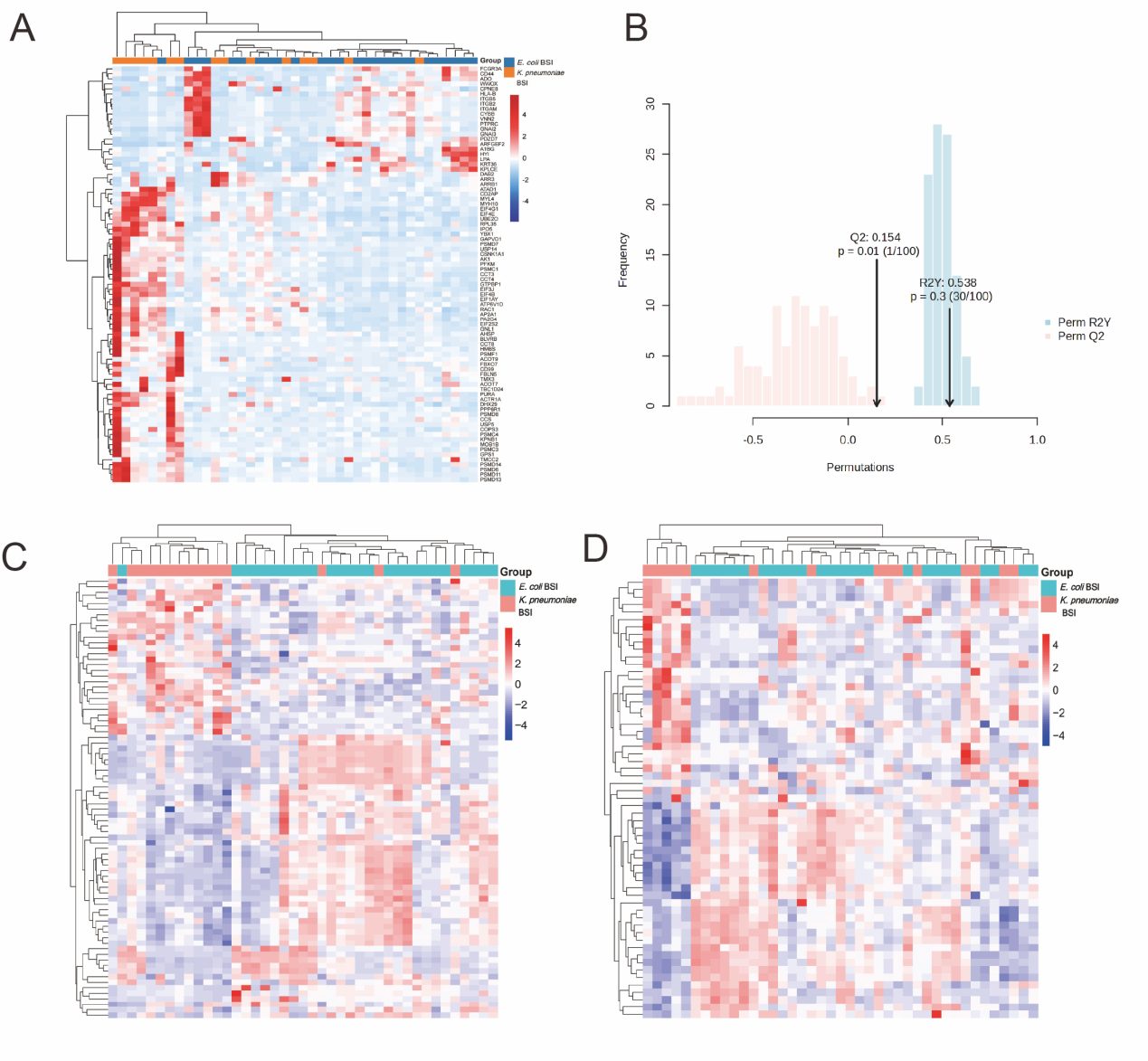
**Figure S5. A,** Heatmap of differential protein expression in the *E. coli* BSI vs *K. pneumoniae* BSI group with 83 proteins before covariate adjustment; **B,** OPLS-DA permutation test for the *E. coli* BSI versus *K. pneumoniae* BSI comparison in positive ion mode, R2Y=0.538, Q2=0.154; **C,** Heatmap of DEM in the *E. coli* BSI vs *K. pneumoniae* BSI group under the POS mode; **D,** Heatmap of DEM in the *E. coli* BSI vs *K. pneumoniae* BSI group under the NEG mode.


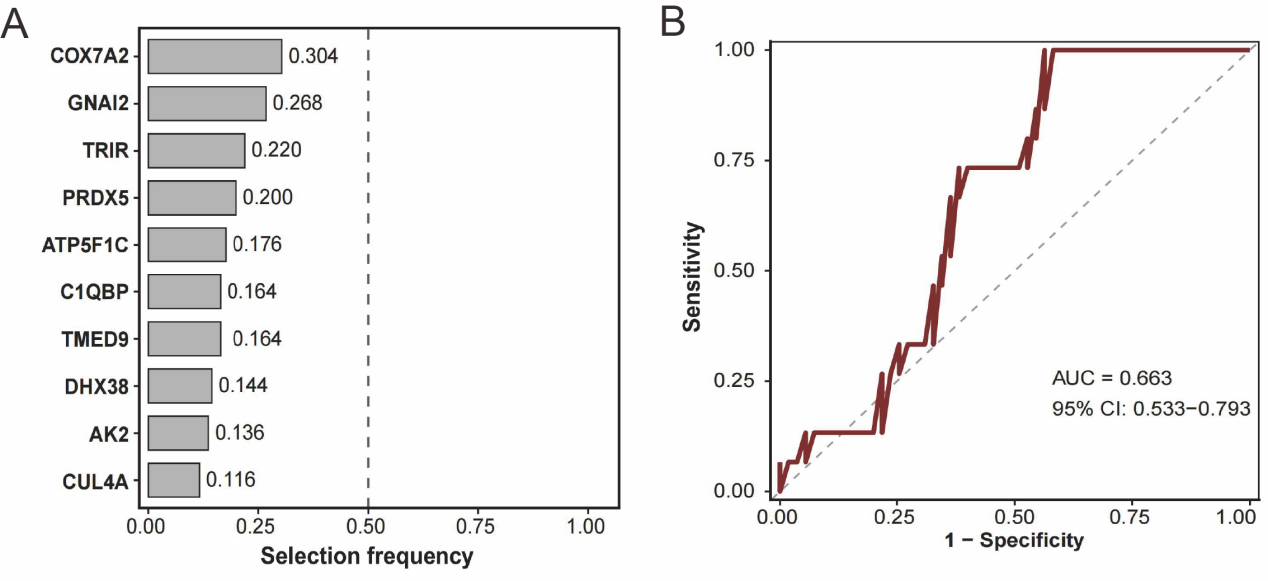


**Figure S6.** Exploratory plasma proteome-based diagnostic model for *K. pneumoniae* BSI. **A**, Feature selection for the exploratory *K. pneumoniae* BSI diagnostic model. **B**, ROC curve for the exploratory model. Because of the small sample size of the *K. pneumoniae* BSI group, this analysis was considered hypothesis-generating and was not used to support clinical translation.

## 2.2 Supplementary Tables

**Table S1**. Stable candidate protein biomarkers and final model coefficients for *E. coli* BSI

| Gene symbol | Accession | Direction in *E. coli* BSI | Selection count | Selection frequency | Coefficient |
| --- | --- | --- | --- | --- | --- |
| FCGR3A | P08637 | Positive direction | 242 | 0.968 | 2.193 |
| COPS3 | Q9UNS2 | Negative direction | 147 | 0.588 | -1.422 |
| NT5C3A | Q9H0P0 | Negative direction | 128 | 0.512 | -3.093 |
| ADO | Q96SZ5 | Positive direction | 125 | 0.500 | 0.634 |

Selection frequency was calculated as the proportion of cross-validation folds in which each protein was retained by LASSO and entered the logistic model. Coefficients were obtained from a final full-cohort logistic regression model for future external validation.

**Table S2**. Internal validation performance of the *E. coli* BSI diagnostic model

| Metric | Value |
| --- | --- |
| Diagnostic task | *E. coli* BSI vs non-*E. coli* sepsis |
| Positive class | *E. coli* BSI |
| Negative class | *K. pneumoniae* BSI + culture-negative sepsis |
| Internal validation | Repeated stratified 5-fold cross-validation |
| Prediction summary | Subject-level averaged held-out predictions |
| No. of patients | 70 |
| No. positive / negative | 26 / 44 |
| AUC (95% CI) | 0.806 (0.706-0.906) |
| Youden-optimized threshold | 0.090 |
| Sensitivity | 1.000 |
| Specificity | 0.523 |
| Accuracy | 0.700 |
| Positive predictive value | 0.553 |
| Negative predictive value | 1.000 |
| Youden index | 0.523 |
| Brier score | 0.186 |

Performance was calculated using subject-level averaged held-out predictions from repeated stratified 5-fold cross-validation. The optimal probability threshold was determined by maximizing the Youden index.
